# Biophysical characterisation of the Bcl-x pre-mRNA and binding specificity of the ellipticine derivative GQC-05: Implication for alternative splicing regulation

**DOI:** 10.3389/fmolb.2022.943105

**Published:** 2022-08-17

**Authors:** Mohammed Bhogadia, Beth Stone, Rafael Del Villar Guerra, Frederick W. Muskett, Sudipta Ghosh, Andrea Taladriz-Sender, Glenn A. Burley, Ian C. Eperon, Andrew J. Hudson, Cyril Dominguez

**Affiliations:** ^1^ The Leicester Institute of Structural and Chemical Biology, Department of Molecular and Cell Biology, University of Leicester, Leicester, United Kingdom; ^2^ The Leicester Institute of Structural and Chemical Biology, Department of Chemistry, University of Leicester, Leicester, United Kingdom; ^3^ Department of Pure Applied Chemistry, University of Strathclyde, Glasgow, United Kingdom

**Keywords:** alternative splicing, G-quadruplex, ellipticine, apoptosis, circular dichroism, NMR

## Abstract

The BCL2L1 gene expresses two isoforms of Bcl-x protein via the use of either of two alternative 5′ splice sites (5′ss) in exon 2. These proteins have antagonistic actions, Bcl-X_L_ being anti-apoptotic and Bcl-X_S_ pro-apoptotic. In a number of cancers the Bcl-X_L_ isoform is over-expressed, resulting in cancer cell survival and growth, so switching splicing to the X_s_ isoform could have therapeutic benefits. We have previously proposed that a putative G-quadruplex (G4) exists downstream of the X_S_ 5′ss and shown that the ellipticine derivative GQC-05, a previously identified DNA G4-specific ligand, induces an increase in the X_S_/X_L_ ratio both *in vitro* and in cells. Here, we demonstrate that this G4 forms *in vitro* and that the structure is stabilised in the presence of GQC-05. We also show that GQC-05 binds RNA non-specifically in buffer conditions, but selectively to the Bcl-x G4 in the presence of nuclear extract, highlighting the limitations of biophysical measurements taken outside of a functional environment. We also demonstrate that GQC-05 is able to shift the equilibrium between competing G4 and duplex structures towards the G4 conformation, leading to an increase in accessibility of the X_S_ 5′ss, supporting our previous model on the mechanism of action of GQC-05.

## 1 Introduction

G-quadruplexes (G4s) are four-stranded Hoogsteen-paired secondary structure elements formed from DNA or RNA sequences with a high density of guanine tracts conforming to the canonical motif G_x_N_y1_G_x_N_y2_G_x_N_y3_G_x_, where x is the number of consecutive guanines (which must be at least 2) and y the number of nucleotides in the connecting loops (Kikin et al., 2006). G4 structures are particularly stable in the presence of monovalent cations, with different cations having variable stabilising effects: Potassium (K^+^) > Rubidium (Rb^+^) > Sodium (Na^+^) > Lithium (Li^+^) or Caesium (Cs^+^) (Sen and Gilbert, 1990). To date, around 300 structures of G4s have been deposited in the PDB, although comparatively few of them are RNA G4s (Banco and Ferre-D'Amare, 2021).

RNA G4s have been implicated in a wide variety of biological processes, such as heterochromatin formation (Takahama et al., 2013), mRNA transcription and 3′ end processing (Dalziel et al., 2007; Zheng et al., 2013), mRNA localization (Subramanian et al., 2011), mRNA translation (Bugaut and Balasubramanian, 2012) and alternative splicing of pre-mRNAs (Marcel et al., 2011; Ribeiro et al., 2015; Huang et al., 2017). However, most of the evidence for the presence of G4s in pre-mRNA mainly comes from two lines of evidence: biophysical experiments with short sequences, taken out of the context of the full-length transcript, in which there might be folding pathways that prevent quadruplex formation, or genetic assays of mutants, which again might be confused by effects on secondary structures.

**FIGURE 1:**
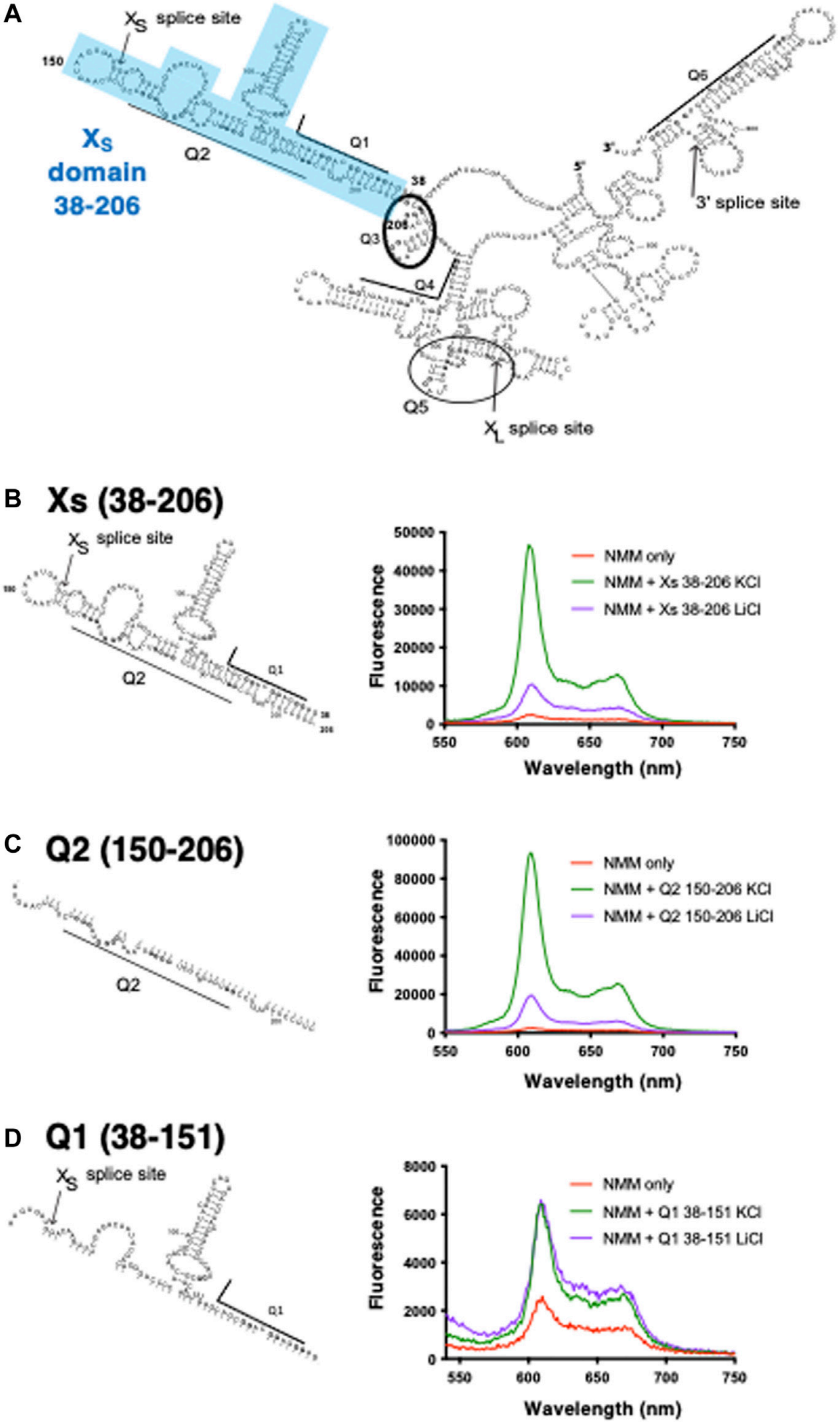
Schematic of RNAs used in this study and NMM fluorescence enhancements in presence of the X_S_ domain and its constituent fragments **(A)** Bcl-x 681 structure previously generated using foot-printing data (adapted from Weldon et al., 2017). Highlighted in blue is the X_S_ domain which covers positions 38–206 containing the Q1 and Q2 PQS and harbours the X_S_ 5’ss. Underlined or circled are the six PQS segments embedded within the RNA structure **(B–D)** NMM emission spectra in the presence of X_S_ 38–206 **(B)**, Q2 150–206 **(C)**, and Q1 38–151 **(D)** in the absence (red) or in presence of 10 µM RNA and 100 mM KCl (green) or LiCl (purple). Experiments were conducted in 10 mM Tris pH 7.0. For clarity, as we are comparing the relative changes in the fluorescence intensity between KCl and LiCl, the *Y* axis scaling is different in each panel.

To address these issues, we developed a novel method, FOLDER (footprinting of long-7-deazaguanine-substituted RNAs), in which guanines are replaced by 7-deazaguanine (Weldon et al., 2017). The use of FOLDER highlighted potential G4-forming sequences adjacent to the alternative 5’ splice sites (5′ss) of the Bcl-x pre-mRNA (Weldon et al., 2017) that give rise to either a pro (X_S_) or an anti (X_L_) apoptotic protein variant (Boise et al., 1993). In particular, we observed that the X_S_ 5′ ss could be sequestered within local secondary structure (Figure 1A), but that formation of the G4 would prevent this and make the X_S_ 5′ ss more accessible to splicing components such as U1 snRNPs (Weldon et al., 2017; Weldon et al., 2018). This model was supported by mutations that would disrupt the secondary structure but not the G4 and others that would restore the secondary structure but disrupt the G4 (Weldon et al., 2018).

These observations suggested to us that the use of the X_s_ 5′ ss would be promoted by ligands that stabilise G4 structures. A small screen of known G4 ligands showed that the ellipticine derivative GQC-05, a previously-reported DNA G4-stabilising ligand (Brown et al., 2011), binds to the Bcl-x pre-mRNA and shifts splicing towards the production of the X_S_ isoform (Weldon et al., 2018). However, other well-described G4 ligands such as carboxyPDS, 360A and PhenDC3 had little effect on Bcl-x splicing (Weldon et al., 2018), suggesting that the mechanisms of this effect were more specific than generic stabilisation of G4s. In view of this, it seemed possible that investigations into the mechanisms of action of GQC-05 might facilitate targeted therapeutic interventions by small molecules that would shift splicing from the predominant X_L_ 5′ ss, the use of which is enhanced in many cancers, towards the Xs 5′ ss. There have been previous reports of strategies that promote shifts in Bcl-X splicing, including the use of inhibitors of SR protein phosphorylation (Moore et al., 2010; Shkreta et al., 2017) and splice switching oligonucleotides (SSOs) that block the X_L_ 5′ ss and thereby reduce the overall cell viability of a variety of cancer cell lines (Mercatante et al., 2002; Bauman et al., 2010; Li et al., 2016). Both of these approaches have drawbacks: alterations in the phosphorylation of SR proteins are likely to lead to widespread changes in splicing, but the intrinsically specific oligonucleotides are likely to perform poorly with solid tumours. Therefore, we have investigated here whether the potential G4-forming region close to the X_s_ 5′ SS does indeed form G4s, and whether GQC-05 can selectively bind this sequence in the presence of nuclear extract. We also provide evidence that the presence of GQC-05 shifts the duplex/G-quadruplex equilibrium in favor of the G-quadruplex, further supporting our initial hypothesis that GQC-05 remodels the Bcl-x pre-mRNA structure and allows higher accessibility of the Xs 5′ ss to the splicing machinery.

## 2 Materials and methods

### 2.1 Chemical synthesis and preparation of short RNA oligonucleotide

Short RNA nucleotides (<30 nucleotides) were purchased in their desalted and deprotected form from Horizon Discovery. RNAs were resuspended in 1 ml milliQ water and dialysed twice in 1 L of milliQ water for 12 h to remove any residual salts and impurities, using a 0.5–1 kDa Spectra-Por dialysis tubing (Cole Parmer, catalogue number: WZ-02906–21). Samples were concentrated using a 3 kDa cut-off Amicon filter (Millipore, catalogue number: MPUFC900308). The concentrations of RNAs were determined by measuring the absorbance at 260 nm at 95°C and using their respective molar extinction coefficients (Supplementary Table S1). RNAs were stored at −20°C. To favor the formation of G4s, the RNA oligonucleotides were incubated at 95°C for 5 min in the desired experimental condition, followed by a slow cooling to room temperature for a minimum of 12 h. RNAs were stored at −20 C.

### 2.2 *In vitro* transcription and purification of RNAs

Long RNAs (>30 nucleotides) were prepared by large scale *in vitro* transcription following optimization of MgCl_2_, NTPs, DNA template and homemade T7 RNA polymerase concentrations. The transcription reactions were centrifuged to remove the pyrophosphate pellet and filtered before injection into a pre-equilibrated anion exchange preparative column (ThermoFisher DNAPac PA100 22 × 250 mm) on an UltiMate 3000 Max HPLC (ThermoFisher), using a 2 ml injection loop with a flow rate of 3 ml/min and a temperature of 80 C. The column was equilibrated with 12.5 mM Tris HCl, pH 7.4, and the RNA was eluted using a gradient of 1 M NaClO_4_ (from 10 to 50% over a period of 60 min) in 12.5 mM Tris HCl, pH 7.4. Relevant fractions were collected, pooled together and then concentrated to approximately 1 ml by centrifugation at 4,000 rpm for 60 min at 4 C using a 3 kDa cut-off Amicon filter (Millipore, catalogue number: MPUFC900308). Samples were then dialysed against 2 L of milliQ water for >12 h using 0.5–1 KDa Spectra-Por dialysis tubing (Cole Parmer, catalogue number: WZ-02906–21), followed by an additional 1 h against 2 L of fresh milliQ water. Aliquots of the samples were then analysed using an 8% denaturing polyacrylamide urea gel run at 180 V for 100 min and stained with a 0.1% toluidine blue solution (in 10% acetic acid) for 5 min to check for RNA purity. The RNA concentrations were determined via A_260nm_ at 95°C.

### 2.3 Circular dichroism

All circular dichroism (CD) experiments were conducted on a Chirascan CD spectropolarimeter (Applied Photophysics). RNA samples, with a concentration of 2 μM, were annealed as described in section 2.1, in 10 mM tetrabutylammonium (TBA) phosphate buffer pH 7.0, with either 100 mM KCl or LiCl. For experiments in nuclear extract, a 5% (v/v) final concentration of HeLa nuclear extract (approximately 1–1.5 mg/ml of nuclear proteins, Cilbiotech, CC-01–20–10) was added to 10 µM RNA post-annealing. Measurements in the absence and presence of nuclear extract were made using a 1 cm and 1 mm pathlength quartz cuvette respectively (total volumes of 1.5 and 0.4 ml respectively), and an integration time of 0.5 s per wavelength value where the final spectrum was obtained by averaging a sequence of three recorded spectra between 200 and 320 nm wavelengths, with a 1 nm step size. The buffer signal was subtracted from the final CD spectrum, including signals emanating from the nuclear extract. CD spectra were normalised relative to the RNA strand concentration. For melting experiments, the spectral data were collected in a stepped ramp mode at 1 °C intervals between 5 and 94°C across a narrower wavelength range of 220–320 nm. CD melting isotherms at 263 nm were normalised, plotted and fitted with either a single or two-state transition model with equations available on GraphPad prism Version 9.0.

### 2.4 NMR spectroscopy

All 1D ^1^H NMR experiments were recorded on a 600 MHz Bruker AV III spectrometer at 283 K using a watergate scheme for suppression of the water signal. Spectra were acquired using 256 scans and an acquisition time of 200 ms with a relaxation delay of 2 s. A Fourier transform was applied using a cosine window function. For titrations with either KCl or GQC-05, a sample of 100 µM RNA was annealed in 10 mM Tris pH 7.0 in the absence of any salt, followed by the addition of D_2_O to a final concentration of 10% (v/v) post annealing. 1D NMR spectra were recorded following successive additions of small volumes of 4 M KCl to final concentrations of 10, 20 and 100 mM. In the case of GQC-05, small volumes of 10 mM GQC-05 (in 100% d_2_DMSO) were added to final concentrations of 100–300 µM.

### 2.5 NMR kinetics and pseudo 2D recordings

100 µM of the Q2 RNA sample was annealed in 10 mM Tris pH 7.0, 20 mM KCl, followed by the addition of 10% D_2_O (v/v) post annealing. An initial 1D spectrum was recorded and then the Q2 complementary strand was added to the Q2 RNA sample at an equimolar concentration, which defined the start of the experiment (t_0_). The acquisition time for each 1D spectrum was 160 s, and the total void time before acquisition of the first spectrum was 562 s. Spectra were recorded at 293 K over a period of 72 h as a pseudo-2D spectrum. This experiment was repeated in the presence of 4 equivalents of GQC-05, which was added to the sample post-annealing of the Q2 strand, followed by addition of the complementary strand. Data were analysed by peak integrals of the imino region (10–14 ppm) of the spectra using Dynamic Centre on Topspin. The kinetic curves of the peak integrals of the imino region versus time were plotted and fitted using first order kinetics with the equation available on GraphPad prism Version 9.0.

### 2.6 Fluorescence spectroscopy

All fluorescence spectra were obtained at 25°C on a Fluromax-4 fluorimeter using a 1 cm pathlength quartz cuvette designed for fluorescence measurements or a Hidex microplate reader on a 384 well plate (Corning) for the fluorescence titrations. For the N-methyl mesoporphyrin (NMM) experiments, 10 µM of the RNAs were annealed in the relevant salt concentrations in 10 mM Tris pH 7.0, followed by the addition of 1 µM NMM. Fluorescence emission spectra were recorded between 450 and 700 nm, with an excitation wavelength of 393 nm and a 5 nm excitation and emission slit width.

For titration experiments using GQC-05 as the fluorophore, all fluorescence titrations were conducted on a Hidex microplate reader using 1 µM GQC-05, with an excitation wavelength of 320 nm and an excitation and emission slit width of 5 nm. 100 µM RNA samples were annealed in 10 mM Tris pH 7.0, 100 mM KCl, followed by the addition of 1 µM GQC-05 post annealing. Concentrations of the RNA were varied through serial dilutions in a buffer containing 10 mM Tris pH 7.0, 100 mM KCl and 1 µM GQC-05, in a total of 17 wells. The final fluorescence emission intensity of the RNA-GQC-05 complex was obtained after subtracting the fluorescence of the individual RNA and GQC-05 components. Data were analysed using GraphPad prism Version 9.0 and fitted to a single-site binding isotherm with the equation reported in (Del Villar-Guerra et al., 2018a).

For fluorescence experiment in presence of nuclear extract, all experiments were conducted as described above, but with the addition of 10% HeLa nuclear extract (v/v) from Cilbiotech post annealing (approximately 2–2.5 mg/ml of nuclear proteins). Background fluorescence arising from interactions between GQC-05 and components of the nuclear extract were subtracted to obtain the final fluorescence signal.

### 2.7 Native gels

10 µM Q2 RNA samples were annealed in 10 mM Tris pH 7.0 with either 100mM KCl, NaCl or LiCl or increasing KCl concentrations (0–20 mM). Samples were loaded on a 12% native PAGE gel at 180 V for 100 min and stained with toluidine blue for 5 min and de-stained in water for >30 min. A mixture of poly(T) DNAs with sizes ranging from 20–120 nucleotides was used as a ladder.

### 2.8 Preparation of Q2 RNA with AlexaFluor546 and DABCYL

A fluorescent dye, AlexaFluor546, and a dark quencher, 4-(dimethylaminoazo)benzene-4-carboxylic acid (DABCYL), were conjugated to modified nucleotides on Q2 RNA located after the first and third G-tracts, respectively (with a 15 base separation). In addition, the 5’ end of Q2 RNA was modified to contain a biotin group. An RNA nucleotide was purchased from Eurogentec with modified thymine bases (X and Y) containing reactive amino and thiol groups, respectively, at the required fluorophore and quencher positions, 5′(Biotin) AAAAAGGGAXGGGGUAAACUGGGGYCGCAUUGUGG AAAAA3’. The A-tracts at the 5 and 3′ ends were included to avoid RNA degradation by nucleases in the nuclear extracts. The amino group on X was subsequently reacted with AlexaFluor546-succinimide (Thermofisher) and the thiol group with DABCYL-maleimide (Eurogentec), respectively. The custom-modified Q2 RNA oligonucleotide (5 μl, 1 mM) was mixed with succinimide-AlexaFluor546 (5 μl, 20 mM) in a microcentrifuge tube, which was protected from light. The tube was placed in a rotary mixer at 2 r.p.m. for 6 h at room temperature. The standard buffer present in G25 columns, which had been stored at 4°C, was replaced with cold phosphate-buffered saline (0.1 M, pH 7.2) and the sample containing dye-labelled Q2 RNA was added to the column. The column was spun for 2 min at 730 g and the eluent was collected, protected from light and stored in 20 μL aliquots at -20°C. The reducing agent tris(2-carboxyethyl)phosphine (TCEP, 1 μl, 100 mM) was added under nitrogen to an aliquot of the dye-labelled Q2 RNA, which was placed in a rotary mixer (2 r.p.m.) at room temperature for 10 min. DABCYL-maleimide in DMSO (1 μl, 10 mM) was subsequently added under nitrogen and mixed at room temperature using the rotary mixer. Again the Q2 RNA was added to a G25 column containing cold buffered saline, which was spun for 2 min at 730 g and the eluent collected, protected from light and stored in aliquots at -20°C.

### 2.9 Single molecule microscopy

Time-resolved single-molecule fluorescence resonance energy transfer (FRET) was measured using total-internal reflection fluorescence (TIRF) imaging. The apparatus is custom-designed using an Olympus IX73P2F inverted microscope frame, with a TIRF objective (UAPON100XOTIRF). The AlexaFluor546 was excited by a 532 nm solid-state laser (Point Source; 15 mW output), and the emission collected by an electron-multiplied charge coupled device (Photometrics). The surfaces of microscope cover glasses were cleaned with piranha solution (3 parts concentrated sulphuric acid and 1 part 30% hydrogen peroxide), and subsequently functionalised by exposure to (3-aminopropyl)triethoxysilane (2% in acetone). Following washing in acetone and deionised water, the slides were assembled to form a fluidic chamber similar to the design previously described (Selvin, 2008). Monolayer coatings of polyethylene glycol were formed on the internal surfaces of the chambers using a 8:1 mixture of polyethylene glycol bis-succinimidyl valerate, MW 5000 (PEG-SVA-5k), and biotinyl-PEG-SVA-5k. A solution of streptavidin (0.2 mg/ml) was added to chambers prior to the addition of the labelled-Q2 RNA, which was added in PBS and became tethered at the biotinylated 5’ end to the PEG surfaces. The imaging buffer comprised 1.5 mM ATP, 20 mM phosphocreatine, 3.2 mM MgCl_2_, 20 mM HEPES-KOH (pH 7.5), 0.05% (v/v) NP40, 50 mM potassium glutamate, 20% nuclear extract (around 4–5 mg/ml of nuclear proteins), 1 mg/ml glucose oxidase, 0.04 mg/ml catalase, 1 mM 6-hydroxy-2,5,7,8-tetramethylchroman-2-carboxylic acid (Trolox), 0.8% (v/v) glucose and, where present, 0.1% (v/v) of 10 mM GQC-05 dissolved in DMSO. Experiments done in the absence of nuclear extract contained an additional 20 mM potassium glutamate to maintain the potassium ion concentration. Images of the emission from surface-tethered molecules of RNA were acquired in frames of 50 ms at ambient temperature.

### 2.10 Electromobility shift assays with U1 oligos

For the WT and optimised (OP) U1 oligo experiments, 150 and 50 µM HPLC-purified X_S_ 38–206 RNA respectively was slow cooled in the absence or presence of 2 equivalents of GQC-05 in 10 mM Tris pH 7.0 and 100 mM KCl. To this, 6 µM of the U1 oligonucleotide (Horizon Discovery) was added post-annealing. The RNA was then serially diluted in an equivalent buffer containing the U1 oligo and with the presence or absence of GQC-05. Samples were loaded on a 16% native polyacrylamide gel, run at 150 V for 1 h and visualised on a Typhoon scanner using an excitation wavelength of 488 nm and detection at 550 nm.

## 3 Results

### 3.1 Characterisation of a G4 within the X_S_ domain of bcl-x by fluorescence turn-on assay

Using a substrate (Bcl-x-681) containing parts of Bcl-X exons 2 and 3 with a shortened intron 2, we identified six candidate G4-forming sequences in Bcl-x-681 that had been labelled as Q1 to Q6 (Weldon et al., 2017) (Figure 1A). Previous work using FOLDER suggested that a G4 is likely to exist around the X_S_ 5′ss (Q2) (Weldon et al., 2017). To confirm the presence of G4s within the X_S_ domain (positions 38–206 of Bcl-x; Figure 1B and Supplementary Table S1), we used the porphyrin derivative N-methyl mesoporphyrin (NMM), since NMM has been shown to bind with high specificity and affinity to parallel G4s, and displays a large fluorescent enhancement when bound to parallel G4s in comparison to anti-parallel or hybrid G4 structures (Nicoludis et al., 2012; Sabharwal et al., 2014; Kreig et al., 2015). We compared the fluorescence enhancement of NMM when incubated with the X_S_ domain and shorter fragments (Figures 1C,D) in the presence of either KCl (a G4 stabiliser) or LiCl (a G4 destabiliser). In the absence of RNA, NMM has a relatively low fluorescence emission as expected (Figures 1B–D, Red), but the fluorescence is significantly increased in the presence of the X_S_ domain and KCl (Figure 1B, Green), demonstrating that NMM binds this RNA. Furthermore, there is an approximately 5-fold increase in the NMM emission in the presence of KCl compared with LiCl (Figure 1B, green and purple), in agreement with the presence of a G4 in the X_S_ domain. This domain contains two putative quadruplex sequences (PQS) that have the potential to fold into a G4: the G-tracts of Q1 which lie upstream of the 5′ss, and the G-tracts of Q2 which lies downstream of the 5′ss (Figure 1A, Supplementary Table S1). Therefore, to identify which region(s) within the X_S_ domain is responsible for the G4 formation and NMM binding, we tested these two halves of the X_S_ domain. For the Q2 150–206 fragment, we observe an approximately 5-fold enhancement of the NMM fluorescence emission in the presence of KCl compared with LiCl (Figure 1C), suggesting that a G4 is present between positions 150–206. Furthermore, the fluorescence enhancement is approximately twice the enhancement observed for the Xs 38–206 region, suggesting that NMM binds with a greater affinity to this shorter RNA, and thus that the Q2 150–206 contains a more stable G4 than Xs 38–206. In contrast, the Q1 38–151 fragment did not show any fluorescence enhancement of NMM in the presence of KCl relative to LiCl (Figure 1D), suggesting that this region does not fold into a G4. Therefore, from this data, we can conclude that the Q2 PQS, but not Q1 PQS, adopts a G4 conformation in the context of the Bcl-x X_S_ domain.

### 3.2 Circular dichroism characterisation of a G4 in the X_S_ domain

**FIGURE 2:**
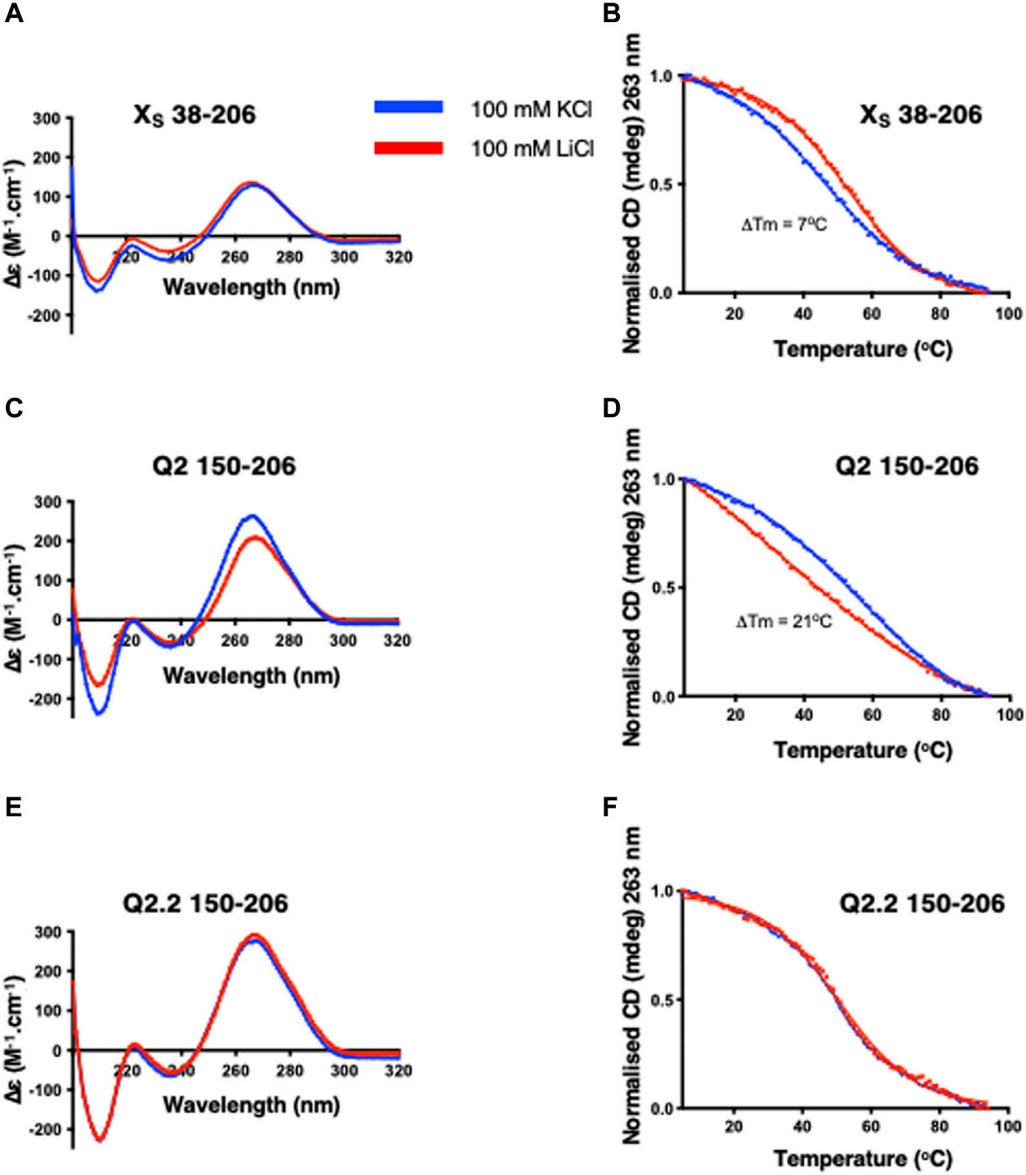
CD spectra and thermal denaturation melting curves of the indicated Bcl-x fragments. CD at 20°C and thermal melting curves in the presence of either KCl (blue) or LiCl (red) of X_S_ 38–206 **(A,B)**, Q2 150–206 **(C,D)** and Q2.2 150–206 **(E,F)**. All experiments were carried out with 2 µM RNA in 10 mM TBA phosphate buffer pH 7.0 with 100 mM salt in a 1 cm pathlength quartz cuvette.

We next investigated whether the Q2 G4 could be present in the X_S_ 38–206 and Q2 150–206 constructs even in the absence of NMM, as NMM may induce a G4 that would otherwise not be present. To do this we compared the CD spectra of the RNAs in the presence of KCl or LiCl in order to distinguish between G4 and duplex structures (Figure 2). For the X_S_ 38–206 domain, containing Q1 and Q2 PQS but also a putative stem-loop structure (Figure 1B), the CD spectra in the presence of KCl or LiCl are similar but not overlapping, with changes occurring at wavelengths between 200 and 250 nm, suggesting small conformational differences of this RNA in KCl and LiCl containing buffers (Figure 2A). Accordingly, the CD thermal melting curves do not overlap in the presence of the two salts, with a lower stability of the X_S_ domain in the presence of KCl compared with LiCl (Figure 2B). The reduction in the melting temperature in the presence of KCl is consistent with the X_S_ domain adopting a duplex structure in LiCl (Figure 1B) that is slightly destabilised in KCl, possibly due to the competition with the G4 formation that we proposed previously (Weldon et al., 2018).

For the Q2 150–206 fragment that contains the Q2 PQS but cannot form the putative stem-loop, the CD spectra show larger differences in the presence of KCl or LiCl buffers (Figure 2C) suggesting significant conformational differences. In agreement, the thermal melting curves show a 21°C increase in the melting temperature in the presence of KCl compared to LiCl (Figure 2D), consistent with the presence of a G4 in this RNA fragment. To confirm that the changes we observe in both the CD spectrum and the CD melting experiments are due to the formation of a G4 structure, we used a G4-abolishing RNA mutant, Q2.2 150–206 (2), which contains a mutation within the Q2 third G-tract, thereby preventing G4 formation (Supplementary Table S1). The CD spectra in KCl and LiCl overlap (Figure 2E) and there are no changes in the thermal stability (Figure 2F), confirming that the changes observed for the Q2 sequence are due to the formation of G4 structures.

### 3.3 CD characterisation of the Q2 G-tracts

**FIGURE 3:**
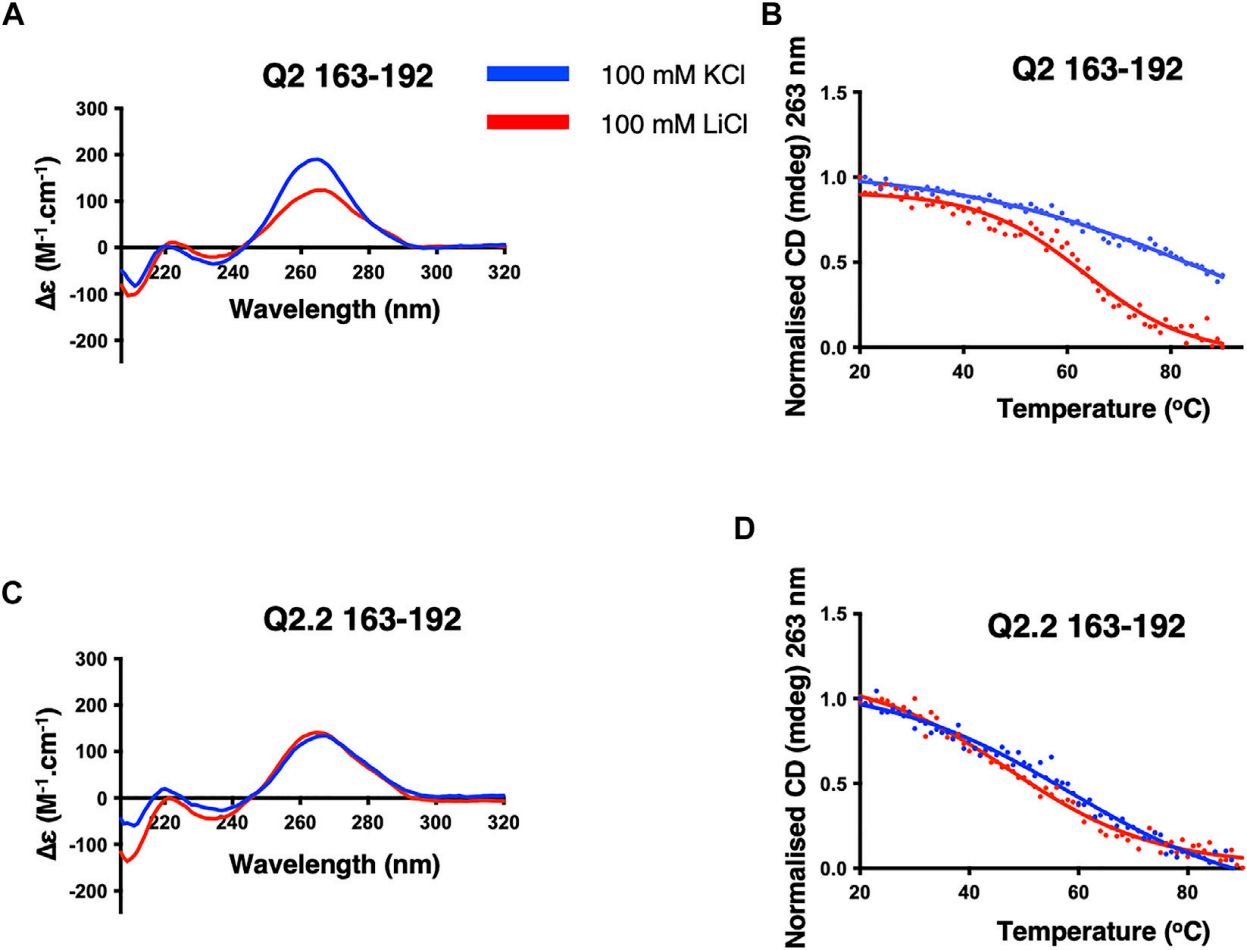
CD spectra and thermal melts of the Q2 and Q2.2 163–192 Bcl-x fragments. CD at 20°C and thermal melting curves in the presence of KCl (blue) and LiCl (red) of Q2 163–192 **(A,B)** and Q2.2 163–192 **(C,D)**. All experiments were conducted with 2 µM RNAs in 10 mM TBA phosphate buffer pH 7.0 with 100 mM salt in a 1 cm pathlength quartz cuvette.

Having shown that a G4 element is likely to form in the G-tracts corresponding to the Q2 region, we next conducted a detailed structural analysis of the short Q2 G-tract within nucleotides 163–192 (Supplementary Table S1). The CD spectrum displays a greater amplitude around 260 nm and a higher thermal stability in KCl than LiCl, similar to the Q2 150–206 RNA, indicating the formation of a parallel G4 conformation (Del Villar-Guerra et al., 2018b) (Figures 3A,B). As before, mutation of the third G-tract in Q2.2 163–192 RNA led to very similar CD spectra and melting temperature in KCl and LiCl. Altogether, these results suggest that the Q2 fragment is forming a stable parallel G4 structure in the presence of potassium.

### 3.4 Q2 adopts multiple higher-order G4 conformations in the presence of K^+^ ions

**FIGURE 4:**
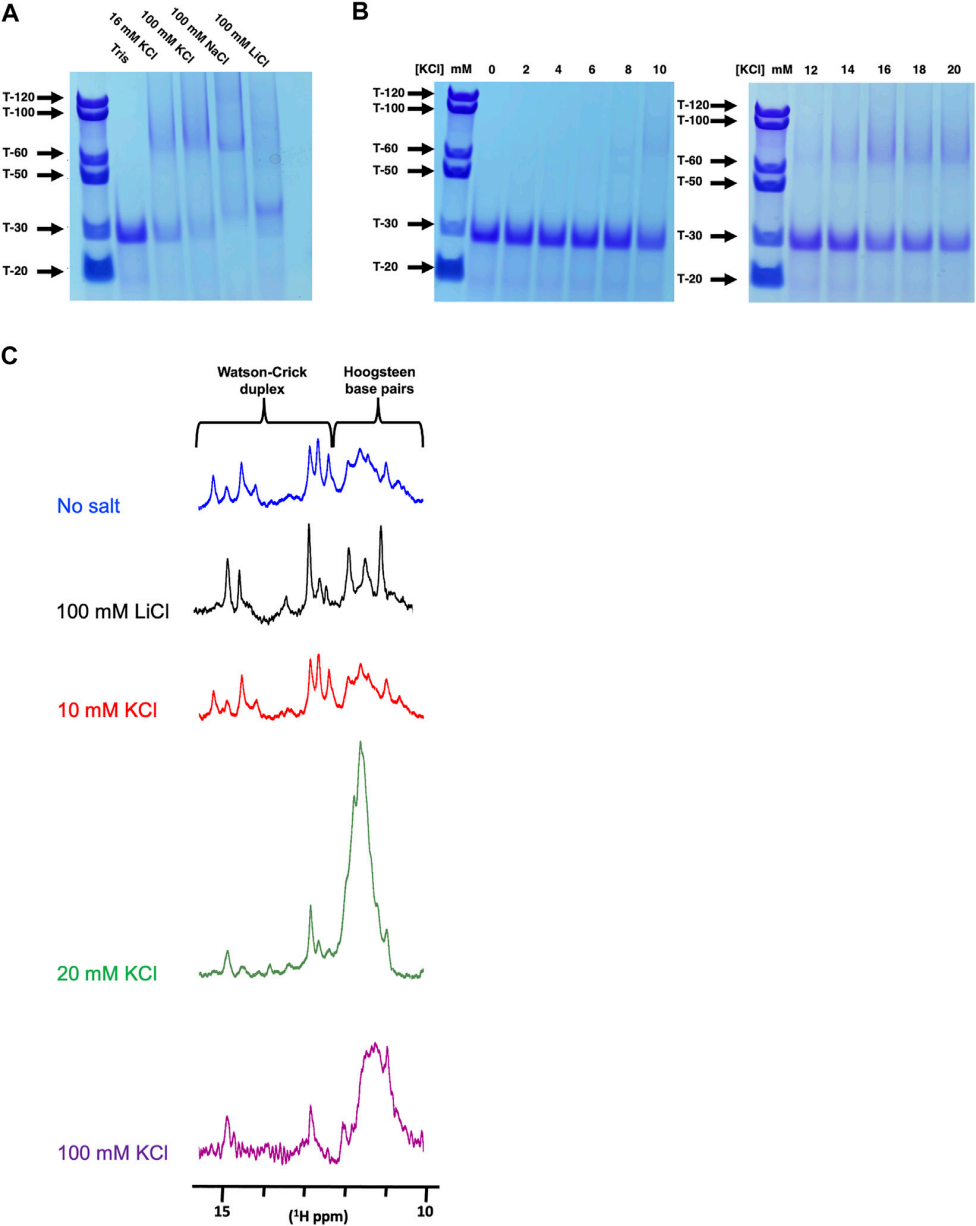
Native PAGE and NMR analysis of Q2 163–192 at different K^+^ concentrations **(A)** 12% Native PAGE of Q2 163–192 RNA following incubation in the presence of Tris pH 7.0, with either no added salt, 16 mM KCl, 100 mM KCl, 100 mM NaCl or 100 mM LiCl. Poly-T of various sizes were used as molecular standards. **(B)** 12% Native PAGE of Q2 163–192 RNA following incubation in the presence of Tris pH 7.0, with increasing concentrations of KCl (0–20 mM) **(C)** ^1^H NMR of the wild-type Q2 163–192 in 10 mM Tris pH 7.0, with no added salt, 100 mM LiCl or various concentration of KCl (10, 20 and 100 mM).

We next investigated whether the Q2 163–192 G4 was intramolecular or intermolecular, using native PAGE after pre-incubation of the RNA with either LiCl, NaCl or KCl. In Tris without salt, we observe a single predominant band that migrates at a position similar to a 30-nucleotide poly-T (T-30), consistent with the expected size of Q2 163–192. In 16 mM and 100 mM KCl we observed the formation of higher order structures migrating at positions around T-60 suggesting the possibility of a G4 dimer. This was also observed in 100 mM NaCl but not in 100 mM LiCl (Figure 4A). We next observed the effect of KCl concentration on the formation of higher order structures. Between 0 and 10 mM KCl, we observe mainly a band corresponding to the monomeric form, while between 12 and 20 mM KCl concentrations, higher order structures are observed (Figure 4B). This becomes more apparent between 14–20 mM KCl, with faint bands also migrating near the T-100 and T-120 marks, suggesting that the Q2 G4 adopts multiple high-order G4 conformations in a KCl concentration-dependent manner. To confirm this observation, we analysed the chemical shifts of imino protons by NMR spectroscopy, since imino protons involved in Watson-crick base pairing typically resonate between 13 and 15 ppm while imino protons involved in Hoogsteen base paring resonate between 10 and 12 ppm (Adrian et al., 2012) (Figure 4C). In the absence or presence of 10 mM KCl or 100 mM LiCl, we observe imino peaks between 10 and 15 ppm suggesting a mixture of duplex and quadruplex conformations. However, in the presence of 20 mM or 100 mM KCl, the intensity of the peaks in the 13–15 ppm region is reduced and a large broad peak appears between 10 and 12ppm, suggesting that the RNA adopts predominantly heterogeneous, probably multimeric G4 conformations. Together with the native PAGE results, we conclude that, in the absence of sequences able to form a competing secondary structure, Q2 RNA adopts a monomolecular G4 conformation at low KCl concentrations (below 10 mM) but multiple higher-order G4 structures at higher KCl concentrations. We also conclude that high KCl concentrations are necessary to increase the general population of G4 structures over any duplex counterparts.

### 3.5 GQC-05 shows greater G4 specificity in the presence of nuclear extract

Knowing that a multimeric G4 element exists within the Q2, we next examined the specificity of GQC-05 for this structure, by determining its binding affinity and re-modelling capabilities.

**FIGURE 5:**
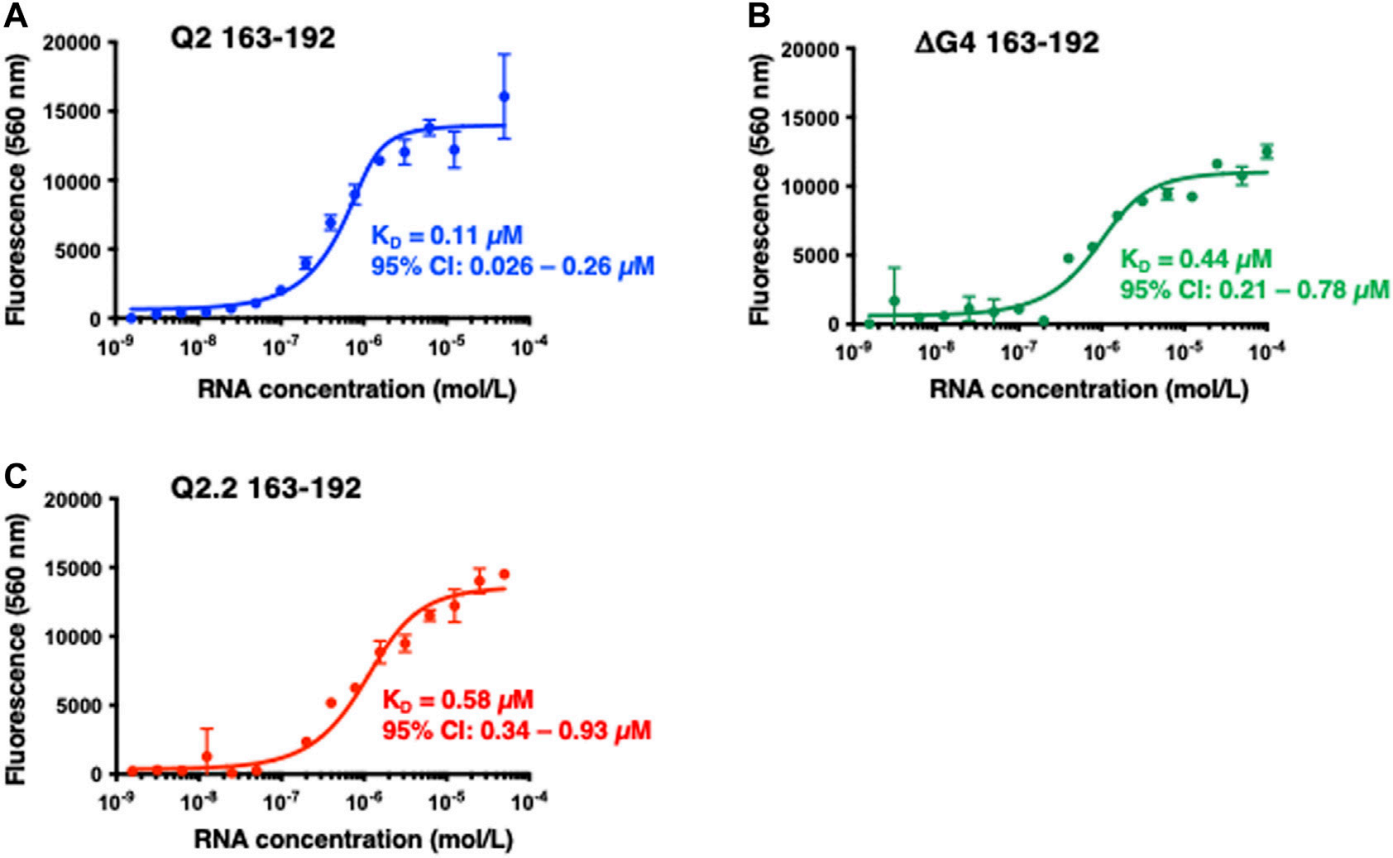
Fluorescence emission at 560 nm (λexc = 320 nm) of GQC-05 as a function of the indicated Bcl-x fragments concentration **(A–C)** Single-site fluorescence binding isotherms of 1 µM GQC-05 incubated with varying concentrations of Q2 163–192 **(A)**, ΔG4 163–192 **(B)** and Q2.2 163–192 **(C)**. Experiments were conducted in 10 mM Tris pH 7.0, 100 mM KCl, at 20°C.

We had previously postulated that GQC-05 can interact with and stabilise the Q2 G4, thereby destabilising a stem-loop structure and ultimately increasing the X_S_ 5′ss usage (Weldon et al., 2018). However, ellipticine molecules have also been described to bind various DNA and RNA structures and it is not clear how specific GQC-05 is for the Bcl-x Q2 RNA. We therefore measured the binding affinity of GQC-05 to Q2 163–192 as well as the non-G4 forming mutants Q2.2 163–192 and ΔG4 163–192 sequences (Supplementary Table S1) using fluorescence spectroscopy at increasing RNA concentrations. The observed dissociation constant (K_D_) for the interaction between GQC-05 and WT Q2 163–192 is 0.11 μM (95% CI: 0.026–0.26 µM) (Figure 5A). Surprisingly, GQC-05 can also bind ΔG4 with a K_D_ of 0.44 μM (95% CI: 0.21–0.78 µM) (Figure 5B) and Q2.2 with a K_D_ of 0.58 μM (95% CI: 0.34–0.93 µM) (Figure 5C); the K_D_ values show that there is only a modest reduction in affinity in comparison with Q2 RNA. This suggests that in the case of RNA, GQC-05 might not be entirely specific for G4s, in contrast to reports with DNA structures (Brown et al., 2011). This is inconsistent with the observation that effects of GQC-05 on splicing are reduced by mutations that would reduce G4 formation (Weldon et al., 2018).

**FIGURE 6:**
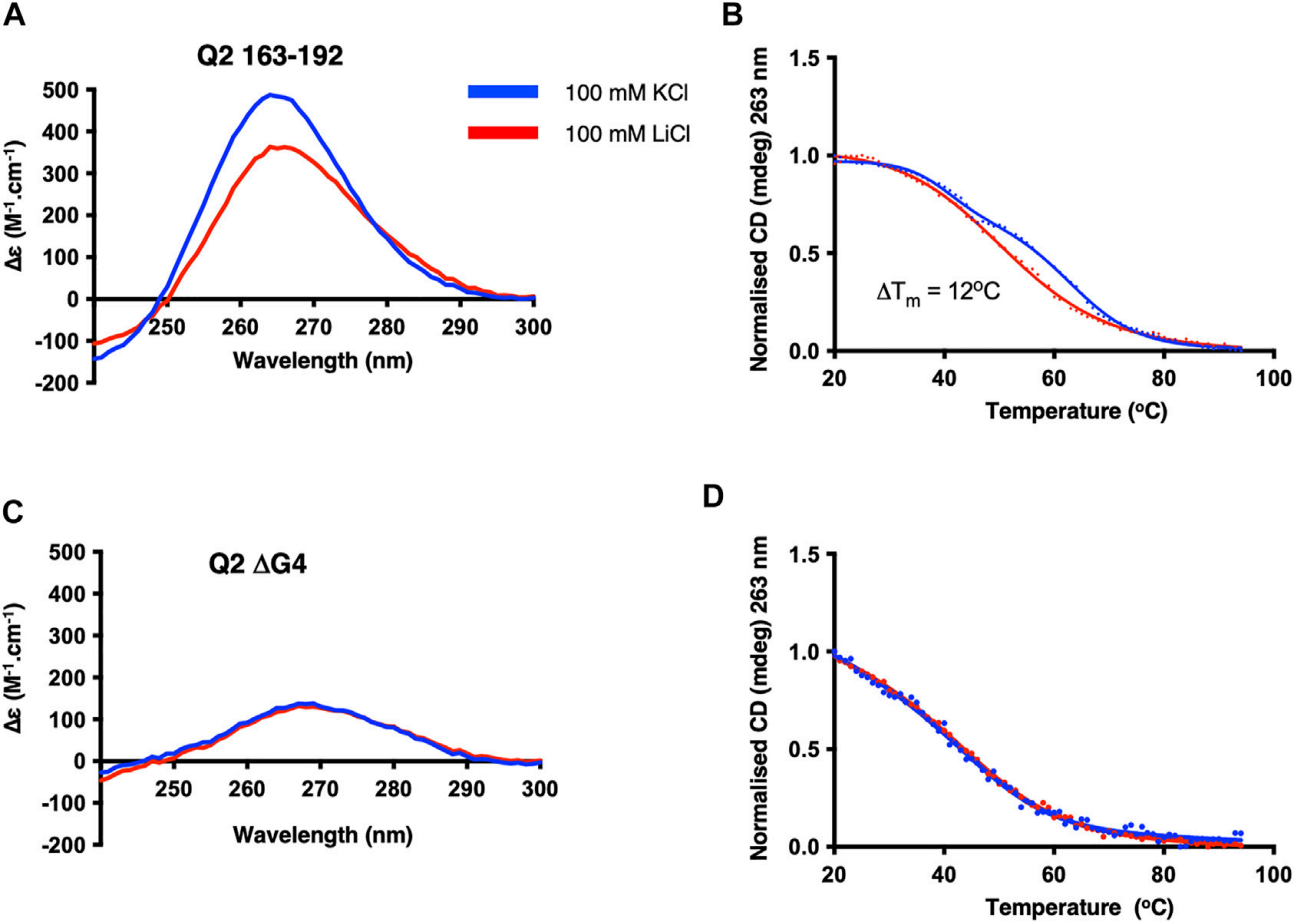
CD structural characterisation of Q2 163–192 and ΔG4 RNA in the presence of HeLa nuclear extract. CD at 20 C and thermal melting curves of Q2 RNA **(A,B)** and ΔG4 RNA **(C,D)**. Experiments were conducted in 10 mM TBA buffer pH 7.0, 5% HeLa nuclear extract, and either 100 mM KCl (blue) or LiCl (red) in a 1 mm pathlength quartz cuvette.

A major difference between the fluorescence assays described above and *in vitro* splicing assays is that fluorescence assays were done with RNA in buffer, while splicing assays are conducted in the presence of nuclear extract containing multiple RNA-binding proteins. We therefore conducted a structural characterisation of the Q2 RNA and investigated the binding of GQC-05 in the presence of HeLa nuclear extract. Even in the presence of nuclear extract, the CD spectra of Q2 163–192 display a greater amplitude as well as a higher melting temperature (ΔTm = 12°C) in 100 mM KCl than in LiCl (Figures 6A,B). In contrast, the ΔG4 mutant did not display any potassium dependence (Figures 6C,D). Interestingly, the thermal stability of the Q2 G4 in the presence of nuclear extract (Figure 6B) is lower than in the absence of nuclear extract (Figure 3B), consistent with the idea that RNA-binding proteins or additional cations present in the nuclear extract may be destabilising the G4 structure.

**FIGURE 7:**
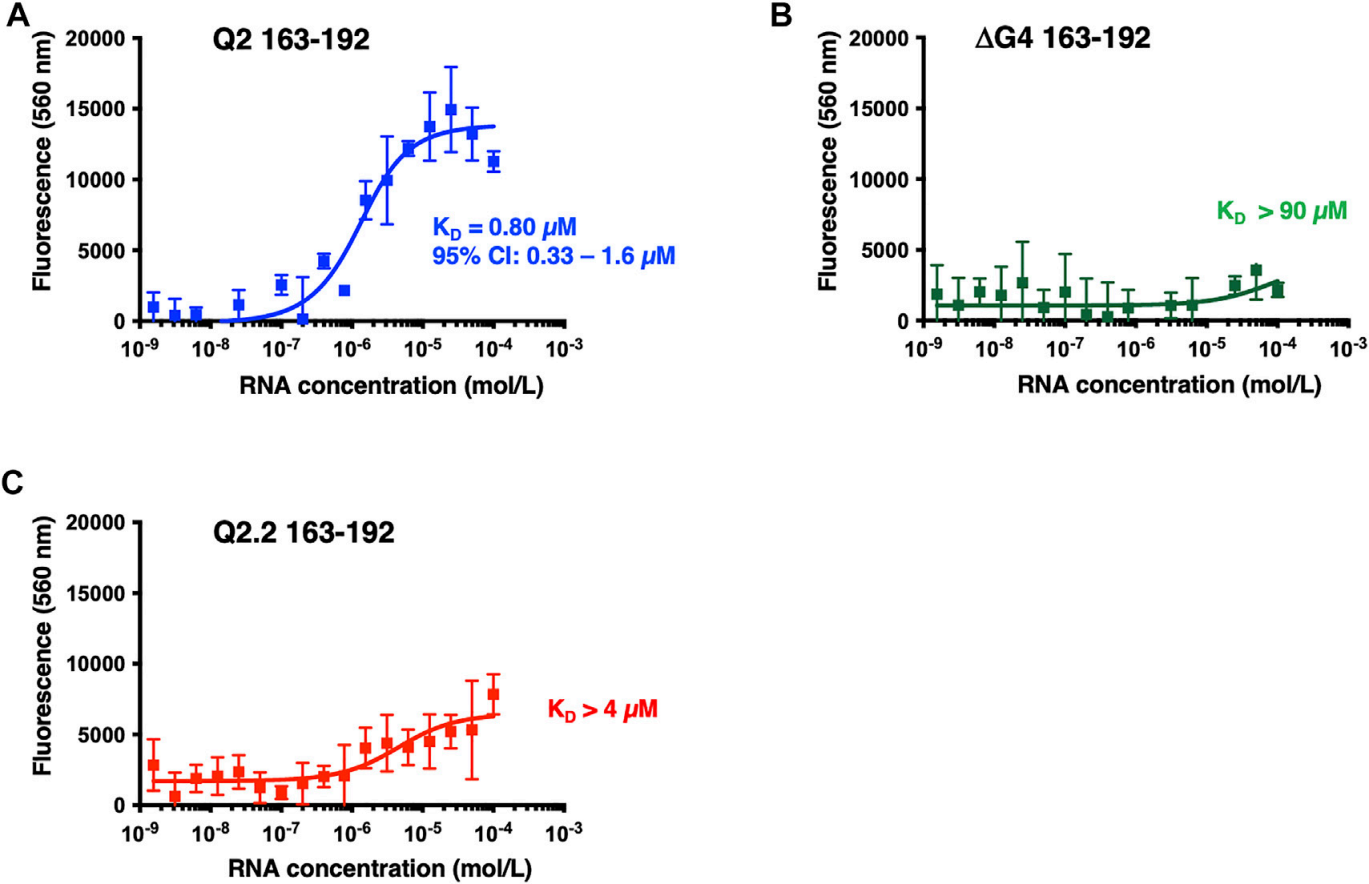
Fluorescence emission at 560 nm (λexc = 320 nm) of GQC-05 as a function of the Bcl-x fragments in the presence of HeLa nuclear extract. Single-site fluorescence binding isotherms of 1 µM GQC-05 incubated with varying concentrations of Q2 163–192 **(A)**, ΔG4 163–192 **(B)** and Q2.2 163–192 **(C)**. Experiments were conducted in 10 mM Tris pH 7.0, 100 mM KCl and 10% HeLa nuclear extract, at 20°C.

Next we investigated the binding of GQC-05 to Q2 163–192 and the Q2.2 and ΔG4 in the presence of nuclear extract (Figure 7). GQC-05 binds the Q2 163–193 RNA even in the presence of nuclear extract, although the affinity is approximately 8-fold lower than in buffer (K_D_ = 0.8 µM, 95% CI: 0.33–1.6 µM) (Figure 7A), consistent with the reduction in stability observed by CD. In contrast, the presence of nuclear extract strongly reduces the affinity of GQC-05 for ΔG4 RNA (K_D_ >90 vs. 0.44 µM, Figures 5B, 7B) and to a lesser extent to Q2.2, which lacks only the central G-tract (K_D_ >4 vs. 0.58 µM,Figures 5C, 7C). Altogether, these results suggest that the presence of nuclear extract confers specificity of GQC-05 for the Q2 163–192 G4 structure, whereas in the absence of nuclear extract both G4 and non-G4 interactions with GQC-05 can take place.

### 3.6 GQC-05 stabilises the Q2 G4 in the presence of nuclear extract

To confirm that the Q2 G4 could be stabilised by GQC-05 in the presence of nuclear extract, we used time-resolved single molecule FRET. We labelled the Q2 RNA after the first G-tract with AlexaFluor546 dye (the donor fluorophore) and after the third G-tract with DABCYL (the acceptor - a dark quencher of fluorescence). AlexaFluor546 and DABCYL will be adjacent in a folded G4 but separated by a greater distance, on average, in the unfolded state of Q2 RNA. This combination of donor/acceptor has a short Föster distance of 2.9 nm, which enables the folding status of Q2 RNA to be monitored sensitively. The unfolded state (non-G4) will exhibit a higher fluorescence photon count for AlexaFluor546, compared with folded RNA (G4) due to reduction in the fluorescence yield from a higher efficiency of resonance energy transfer to the DABCYL quencher.

**FIGURE 8:**
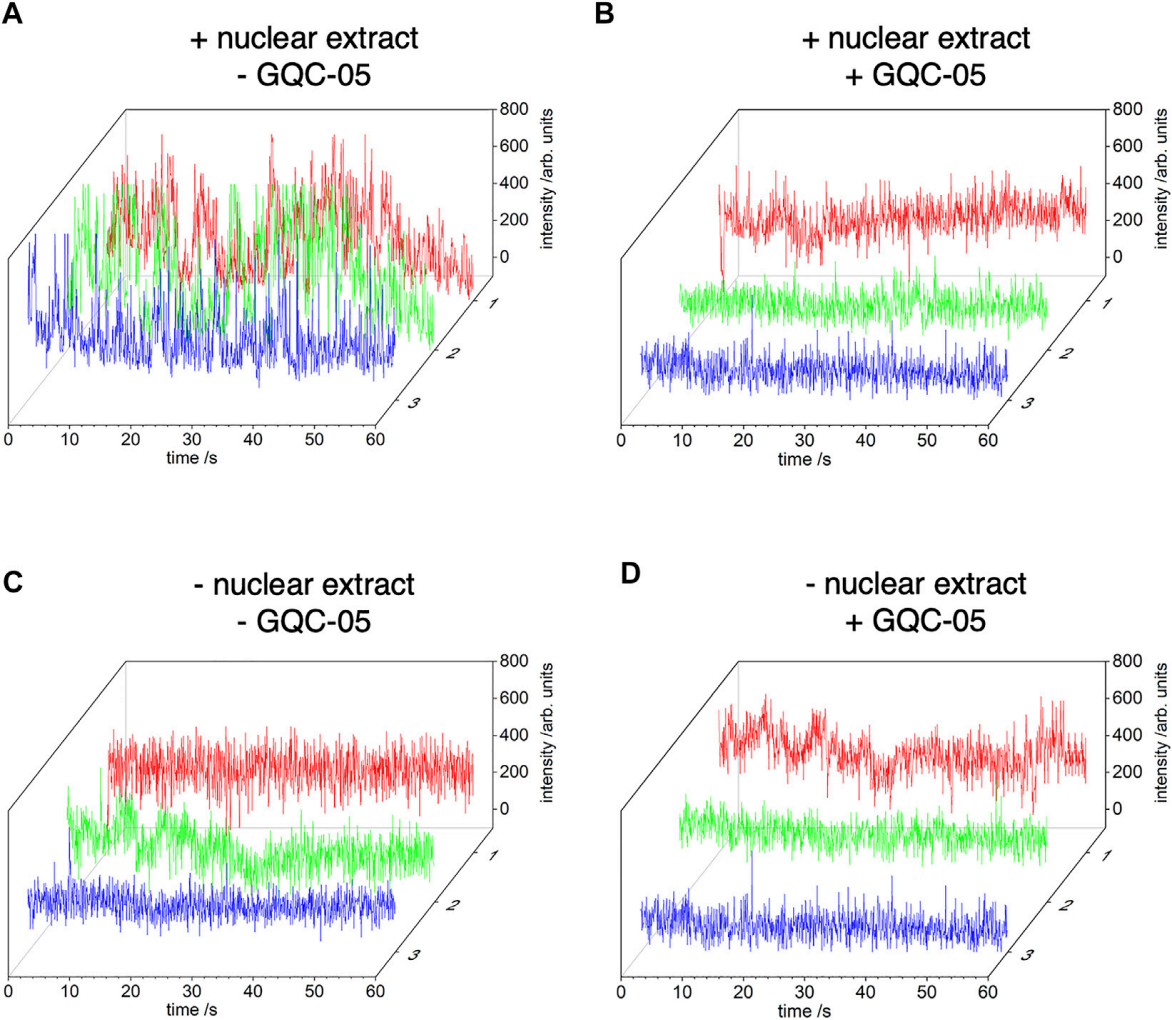
Time-resolved single-molecule (FRET) between AlexaFluor546 and DABCYL. Q2 RNA was labelled, after the first G-tract, with AlexaFluor546 dye and, after the third G-tract, with DABCYL. A biotin-tag was incorporated at the 5′ end of Q2 RNA and the oligonucleotide was tethered to the surface of cover glasses via biotin-avidin linkages. The photon count from AlexaFluor546 is shown for three separate copies of single molecules of Q2 RNA obtained from images recorded with a 50 ms integration time (approx. 20 frame per second). Experiments were conducted in 20% nuclear extract (the exact buffer composition is described in the method section 2.9), in the absence **(A)** or presence **(B)** of GQC-05 respectively. Control experiments were conducted in the same conditions but in the absence of nuclear extract **(C)** and **(D)**.

In the presence of nuclear extract, there are significant temporal fluctuations in the photon count from the Alexa546 dye in the absence of GQC-05 (traces from three representative molecules are shown in Figure 8A) but there is stable emission at a relatively lower intensity level in the presence of GQC-05 (Figure 8B). These results indicate that the Alexa546 dye and DABCYL quencher are located in close proximity in the presence of GQC-05 in nuclear extracts, which is most likely the result of stabilisation of the G4 folded state, whereas the unfolded state is more likely to exist in the absence of GQC-05 in nuclear extracts. This observation is consistent with the results from CD that suggest a transient and polymorphic nature to the Q2 G4 structure in the presence of nuclear extract. As a control, we performed the same experiment in the absence of nuclear extract. As expected, there is a relatively low level of stable fluorescence emission from the AlexaFluor dye, both in the absence and presence of GQC-05, demonstrating that in the absence of nuclear extract, Q2 adopts a G4 structure independently of the presence of GQC-05 (Figures 8C,D).

### 3.7 GQC-05 induces and stabilises the Q2 G4 conformation over its duplex counterpart

**FIGURE 9:**
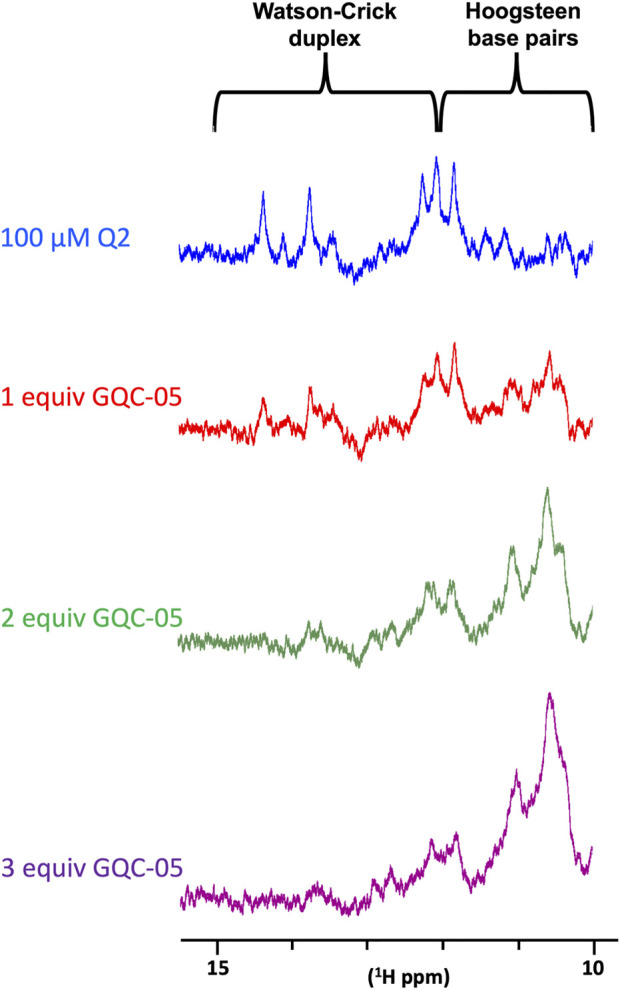
NMR titrations of Q2 163–192 RNA with increasing concentrations of GQC-05.1D NMR spectra of 100 µM Q2 163–192 in the absence or presence of increasing amount (1, 2 and 3 molar equivalent) of GQC-05.

We next examined the effects of GQC-05 on the Q2 163–192 G4/duplex conformational switch. The NMR spectrum of Q2 163–192 in the absence of KCl (Figure 4B) suggests that the RNA adopts a mixture of both G4 and duplex structures, implying the presence of two structural populations in these conditions. However, increasing the KCl concentration induced a shift in the equilibrium towards a G4 structure (Figure 4B). We therefore examined whether GQC-05 could have a similar G4-stabilising effect as KCl. Addition of GQC-05 resulted in a decreased intensity of the imino peak corresponding to Watson-Crick base pairing (13–15ppm), and an increase in intensity of the peaks corresponding to G4 (10–12ppm) (Figure 9) in a dose-dependent manner, remarkably similar to that observed with KCl. This demonstrates that GQC-05 can elicit a conformational change of Q2 by shifting the equilibrium from a G4/duplex mixed population towards the stabilisation of a G4 structure.

**FIGURE 10:**
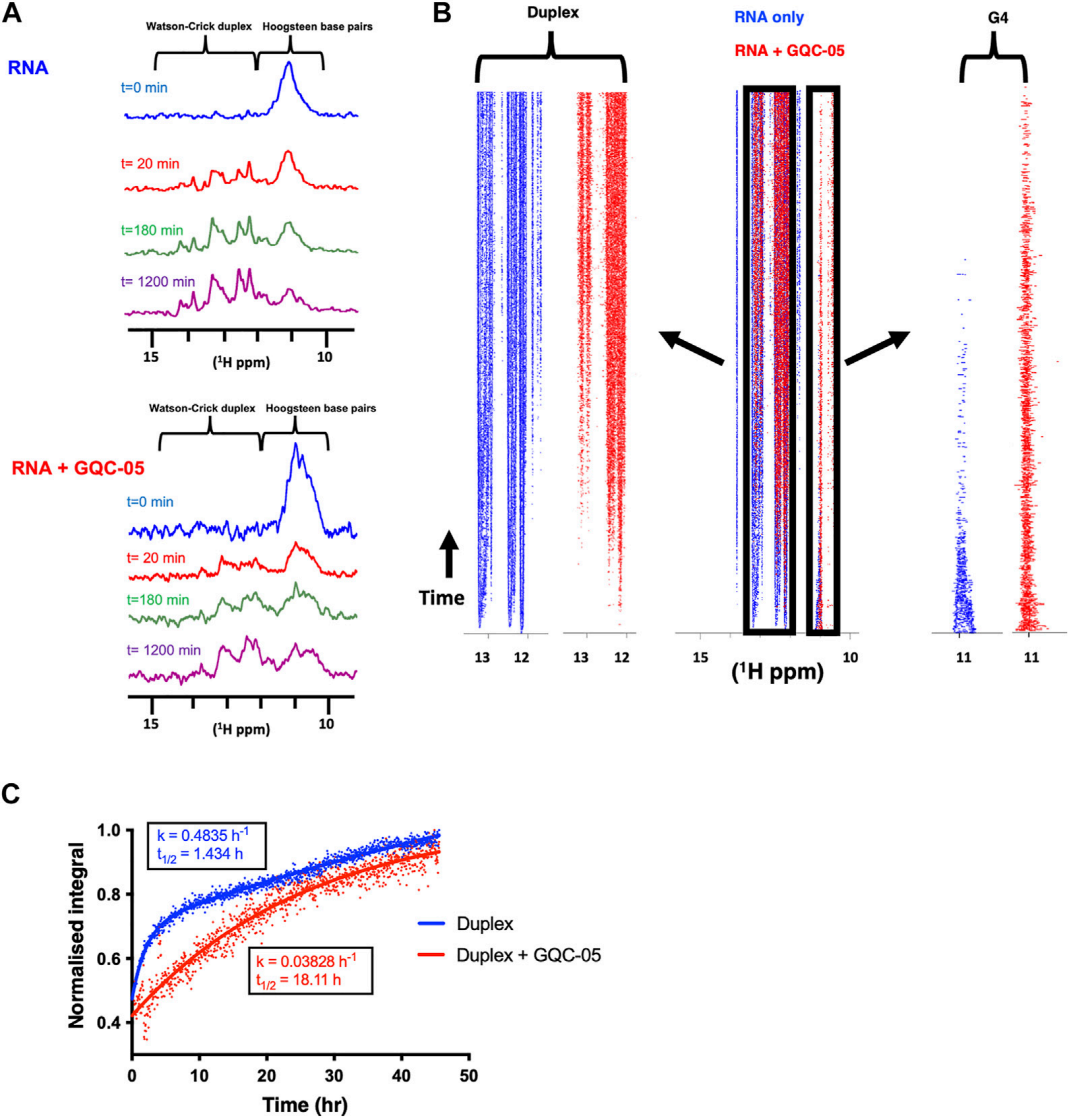
GQC-05 stabilises a G4 over duplex formation of the Q2 163–192 RNA **(A)** ^1^H NMR spectrum at various time points after the addition of a complementary strand in the absence (top) and presence (bottom) of 4 equivalents of GQC-05 **(B)** Pseudo-2D traces displaying the peak intensities across the imino region as a function of time in the absence (blue) and presence (red) of GQC-05. Zoomed in on the left and right panels are the peaks corresponding to the duplex and G4 regions respectively. **(C)** Normalised peak integrals in the duplex region as a function of time and rates fitted to a first order reaction. Experiments were carried out using 100 µM RNAs at 283 K in 20 mM KCl, 10 mM Tris pH 7.0.

To investigate further whether GQC-05 acts by stabilising G4 structures rather than increasing their rate of formation or destabilising duplexes, we performed complement-trapping NMR kinetic experiments, as described previously (Gray et al., 2019). For this, we first measured a 1D NMR spectrum of the Q2 163–192 RNA in conditions that support the formation of a multimeric G4 with low population of duplex species (10 mM Tris pH7, 20 mM KCl) (Figure 10A). This was followed by the addition of an RNA sequence complementary to Q2 163–192 (at an equimolar concentration) and measurement of 1D NMR spectra over a time period of 72 h. The same experiment was then repeated in the presence of 4 molar equivalents of GQC-05. Therefore, by comparing the rate at which the G4s dissociate and the duplex forms in the presence and absence of GQC-05, we can determine whether GQC-05 preferentially stabilises the G4 structure.

After addition of the complementary strand, both in the absence and presence of GQC-05, we observed in a time-dependent manner the expected appearance of imino proton signals corresponding to a duplex and the disappearance of the G4 signals (Figure 10A). The differences in rate of formation of the duplex and dissociation of the G4 were visualised using a pseudo-2D trace (Figure 10B) that displays the peak intensities across the imino regions (10–15 ppm) as a function of time. Analysis of the trace shows that the peaks corresponding to a G4 (10–12ppm) disappear more rapidly in the absence (blue) than in the presence of GQC-05 (red). Accordingly, the imino signals typical of Watson-Crick base-pairs (13–15 ppm) appear almost instantly in the absence of GQC-05 (blue), but much later in the presence of GQC-05 (red).

We then calculated the rates of duplex formation by integrating the peaks in the 13–15 ppm region using first order kinetics (Figure 10C). The estimated half-time (t_1/2_) of duplex formation in the absence of GQC-05 is 1.4 h, whereas it is > 18 h in the presence of GQC-05 at 283 K. These data clearly indicate that GQC-05 can bind to and selectively stabilise the Q2 G-quadruplex structure over the duplex in the Bcl-x Q2 sequence.

### 3.8 GQC-05 increases the accessibility of the X_S_ 5′ss

**FIGURE 11:**
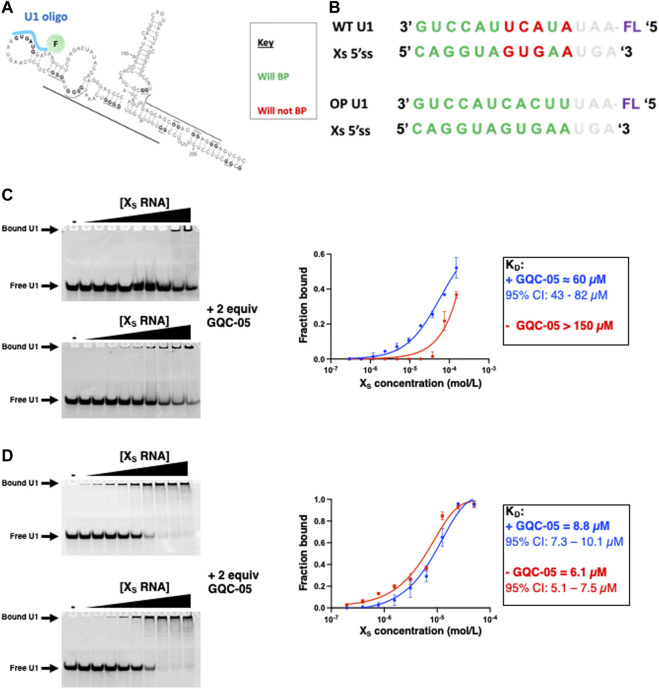
Probing the X_S_ 5′ss accessibility using fluorescently labelled U1 oligos **(A)** Schematic showing the proposed binding of the fluorescently labelled U1 oligo (cyan) **(B)** Sequences of the U1 WT and OP (optimised) oligo and their ability to base pair the X_S_ 5’ss **(C–D)** EMSA assays with a fluorescein labelled wild-type **(C)** and optimised **(D)** U1 oligo at 6 µM as a function of the X_S_ RNA concentration in the absence (Top gel) and presence (Bottom gel) of 2 equivalence of GQC-05 in 10 mM Tris pH 7.0 and 100 mM KCl. Samples were loaded on a 16% native polyacrylamide gel, ran at 4°C at 150 V for approximately 60 min. Gels were visualized on a Typhoon scanner with a 488 nm excitation and 550 nm emission wavelength. The binding isotherms are plotted on the right for the X_S_ 5′ss in the absence (red) and presence (blue) of GQC-05.

Having shown that GQC-05 can bind and favour the formation of a G4 structure over a duplex, we next examined whether such stabilisation could lead to changes in the accessibility of the X_S_ 5′ss. To examine this, we probed the binding of a small fluorescently labelled RNA oligonucleotide that would mimic the base pairing of U1 snRNP to 5′ss (as depicted in Figure 11A) using electrophoretic mobility shift assays (EMSA). The Xs 5′ss is a weak 5′ss splice site because its sequence is not entirely complementarity to human U1 snRNA (Figure 11B).

In the absence of GQC-05, the affinity of the oligonucleotide to the Bcl-X_s_ (38–206) construct is rather weak (K_D_ > 150 µM) suggesting that the formation of the duplex indeed reduces the accessibility of the Xs 5’ss. However, the addition of two molar equivalents of GQC-05 significantly increases its affinity for Bcl-X_s_ (38–206) (K_D_ ≈ 60 μM, 95% CI: 43–82 µM) (Figure 11C), in agreement with our hypothesis that GQC-05 stabilisation of a G4 in the Q2 region induces a destabilization of the duplex and therefore an increase in the splice site accessibility and hence usage of the X_S_ 5′ss. This is also consistent with our previous splicing assays showing that GQC-05 leads to an increase of the X_S_ splice site usage (Weldon et al., 2018). To investigate whether the observed effect of GQC-05 on the U1/5′ss interaction correlates with the weak complementarity of the X_S_ 5′ss to U1 snRNA, we tested another oligonucleotide that is 100% complementary to the X_S_ 5′ss (U1 OP in Figure 11B). In this case, as expected, this oligonucleotide has a stronger affinity to the Bcl-X_s_ (38–206) (K_D_ = 6.1 µM, 95% CI: 5.1–7.5 µM) indicating that the duplex is not stable enough to complete with its binding and as expected, addition of GQC-05 has no significant effect on the affinity (K_D_ = 8.8 µM, 95% CI: 7.3–10.1 µM) (Figure 11D). This suggests that changes in 5′ss accessibility in the presence of GQC-05 are only mediated when the splice site is relatively weak compared to a consensus 5′ss.

## 4 Discussion

We have previously suggested that the Bcl-x pre-mRNA contains two regulatory G4s, termed Q2 and Q5 (Weldon et al., 2017) and showed that the molecule GQC-05 could modulate the splicing of Bcl-x possibly through binding these G4s (Weldon et al., 2018). However, the existence of these G4s and the effect of GQC-05 on their formation were not investigated in detail. Here, using biophysical approaches, we have characterized the structural features of the Q2 G-tract that lies downstream of the X_S_ 5′ss Bcl-x. GQC-05 was previously shown to selectively bind DNA G4 structures over their double stranded counterparts *in vitro* (Brown et al., 2011). However, in this study, we demonstrate that, *in vitro*, GQC-05 binds with similar affinities to both G4 RNA molecules and RNA molecules that are unable to fold into G4 structures, suggesting that GQC-05 does not display selectivity for G4 RNAs. However, surprisingly, we demonstrate that the specificity of GQC-05 towards G4 is achieved in the presence of HeLa nuclear extracts. To our knowledge, this is the first evidence that a ligand displays RNA G4 selectivity only in the presence of nuclear extract. This highlights the limitations of many experiments when determining binding specificities in highly purified *in vitro* systems, without taking into consideration the cellular environment, where the RNA is bound by multiple RNA-binding proteins and the true specificity of a G4 ligand is likely to be revealed. This could be true for many other G4-binding molecules that are known to display a high affinity but low specificity towards G4s in *vitro* conditions. The exact mechanism by which nuclear extract confers GQC-05 specificity to the Q2 G4 structure remains to be explored, though the potential for RNA-binding proteins to compete with non-G4 interactions is a likely possibility.

Even though GQC-05 lacks specificity for G4s in the absence of nuclear extract, our NMR data show that it induced and stabilised the formation of G4 structures over their duplex counterparts (Figures 9, 10). This effect was not seen in the single molecule FRET results, which showed no apparent change in the absence of nuclear extract (Figure 8). This apparent discrepancy is likely to be due to differences in the time scales or dimensions of the motions detected by the two methods. However, the FRET data support the findings that the G4 is unstable in the presence of nuclear extract unless it is stabilised by GQC-05 binding. The bias towards G4 formation induced by GQC-05 supports our initial hypothesis that GQC-05 can stabilise the G4 structure in Q2 at the expense of a long stem-loop (Weldon et al., 2018).

In our previous work, we suggested that the competition between G4 and stem-loop formation could affect the accessibility of the X_S_ 5′ss to potential *trans* acting factors, such as the spliceosomal component U1 snRNP. Indeed our previous structural model of Bcl-x suggested that in the absence of Q2 G4, the Bcl-X_s_ portion of the RNA would adopt a long stem-loop that encompasses part of the Xs 5′ss, making it poorly accessible to the splicing machinery. By probing the binding capability of a U1 snRNA-mimicking oligo to a longer portion of RNA, that contained the sequences required to form the stem-loop, we show that GQC-05 can indeed increase the accessibility of the X_S_ 5′ss, consistent with the observed enhancement in its usage in splicing assays, fully supporting our previously described model (Weldon et al., 2018). However, an oligo that is 100% complementarity to the X_S_ 5′ss binds strongly and the addition of GQC-05 shows no difference in accessibility, indicating that the stability this secondary structure would not be sufficient to regulate splicing if the 5′ss were a consensus sequence, perfectly complementary to the U1 snRNA. Thus, our model for regulation of this site by competition between the formation of secondary structure or a G4 would only hold in circumstances where, as with Bcl-Xs, the 5’ ss is intrinsically weak.

## Data Availability

The raw data supporting the conclusions of this article will be made available by the authors, without undue reservation.
