# Supplementary material for: Biophysical characterisation of the Bcl-x pre-mRNA and binding specificity of the ellipticine derivative GQC-05: Implication for alternative splicing regulation
Source: Front Mol Biosci. 2022 Aug 17;9:943105. doi: 10.3389/fmolb.2022.943105 (PMC9428248; doi:10.3389/fmolb.2022.943105)
Supplement: Supplementary file 1 [file Table1.PDF]

Bcl-x fragment	Sequence	$A_{260\ nm}$ molar absorptivity ($l\ mol^{-1}cm^{-1}$)
**X_S 38-206**	GAGACUGAGGGAGGCAGGCGACGAGUUUGAACUGCGGUACCGGCGGGCAUUCAGUGACCUGACAUCCCAGCU CCACAUCACCCCAGGGACAGCAUAUCAGAGCUUUGAACAGGUAGUGAAUGAACUCUUCCGGGAUGGGGUAAA CUGGGGUCGCAUUGUGGCCUUUUUCUCCUUC	1708500
**Q1 38-151**	GAGACUGAGGGAGGCAGGCGACGAGUUUGAACUGCGGUACCGGCGGGCAUUCAGUGACCUGACAUCCCAGCU CCACAUCACCCCAGGGACAGCAUAUCAGAGCUUUGAACAGGUAGUGAA	1189400
**Q2 150-206**	GAGACUAAUGAACUCUUCCGGGAUGGGGUAAACUGGGGUCGCAUUGUGGCCUUUUUCUCCUUC	608900
**Q2.2 150-206**	GAGACUAAUGAACUCUUCCGGGAUGGGGUAAACUGUUGUCGCAUUGUGGCCUUUUUCUCCUUC	608100
**Q2 163-192**	GGGAUGGGGUAAACUGGGGUCGCAUUGUGG	303400
**Q2.2 163-192**	GGGAUGGGGUAAACUGUUGUCGCAUUGUGG	302600
**ΔG4 163-192**	GUGAUGUUGUAAACUGUUGUCGCAUUGUGU	301000

Table S1: Bcl-x fragments used in this investigation for biophysical analysis and their respective molar extinction coefficients.
